# Supplementary material for: Analysis of Post-Transplant Lymphoproliferative Disorder (PTLD) Outcomes with Epstein–Barr Virus (EBV) Assessments—A Single Tertiary Referral Center Experience and Review of Literature
Source: Cancers (Basel). 2021 Feb 21;13(4):899. doi: 10.3390/cancers13040899 (PMC7924879; doi:10.3390/cancers13040899)
Supplement: Supplementary file 1 [file cancers-13-00899-s001.zip › Table S1 - Clinical Characteristics of M-DLBCL-PTLD patients with EBV-VL based on the 100000 copies mL cutoff.pdf]

Table S1: Clinical Characteristics of M-DLBCL-PTLD patients with EBV-VL based on the 100,000 copies/mL cutoff

Variables	Subtypes	>100,000 copies/mL	≤100,000 copies/mL
Total		5	19
Sex	Male	3 (60%)	11 (58%)
	Female	2 (40%)	8 (42%)
Transplant Age	Infant	1 (20%)	5 (26%)
	Pediatric/Adolescent	2 (40%)	7 (37%)
	Adult	2 (40%)	7 (37%)
Transplanted Organ	Heart	4 (80%)	11 (58%)
	Kidney	1 (20%)	6 (32%)
	Liver	0 (0%)	2 (11%)
Time to PTLD Diagnosis	Early	1 (20%)	3 (16%)
	Late	4 (80%)	10 (53%)
	Very Late	0 (0%)	6 (32%)
ECOG Status	0-2	3 (60%)	13 (68%)
	3-4	2 (40%)	6 (32%)
CD20 Status	Positive	5 (100%)	17 (89%)
	Negative	0 (0%)	1 (5%)
	Unknown	0 (0%)	1 (5%)
Tumor EBER Status	Positive	5 (100%)	16 (84%)
	Negative	0 (0%)	3 (16%)
Treatment Response	Progressive Disease	1 (20%)	6 (32%)
	Partial Response	0 (0%)	1 (5%)
R-IPI Score	Complete Response	4 (80%)	12 (63%)
	Low (0-2)	2 (40%)	13 (68%)
	High (3-4)	3 (60%)	6 (32%)
