# Supplementary figures and images for: Analysis of Post-Transplant Lymphoproliferative Disorder (PTLD) Outcomes with Epstein–Barr Virus (EBV) Assessments—A Single Tertiary Referral Center Experience and Review of Literature
Source: Cancers (Basel). 2021 Feb 21;13(4):899. doi: 10.3390/cancers13040899 (PMC7924879; doi:10.3390/cancers13040899)

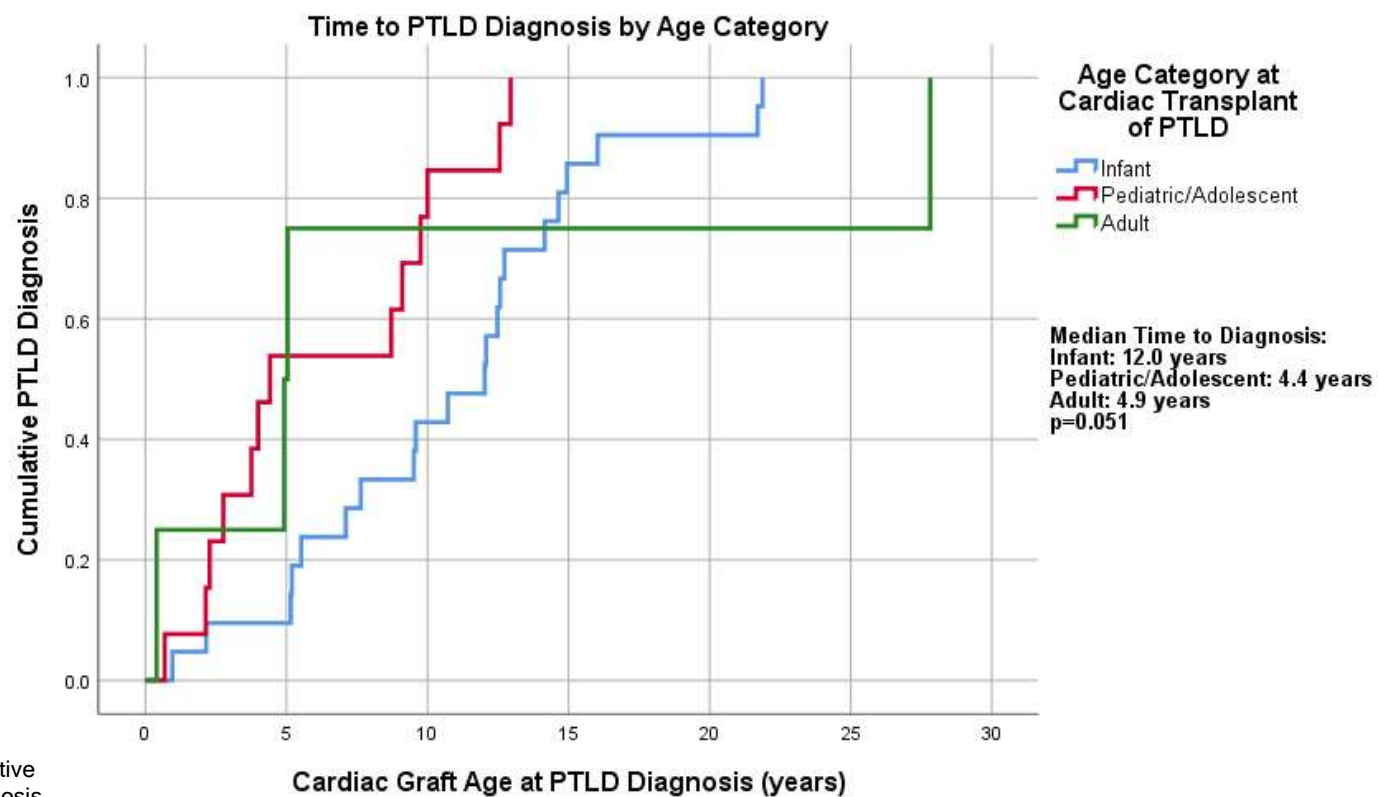

# of Cumulative  
PTLT Diagnosis

|                      |   |   |    |    |    |    |   |
|----------------------|---|---|----|----|----|----|---|
| Infant               | 0 | 2 | 9  | 18 | 19 | 21 |   |
| Pediatric/Adolescent | 0 | 7 | 10 | 13 |    |    |   |
| Adult                | 0 | 2 | 3  | 3  | 3  | 3  | 4 |

Supplement: Supplementary file 1 [file cancers-13-00899-s001.zip › Figure S1 - Time to PTLD by Age Category, Including only Cardiac Transplant Recipients.pdf]

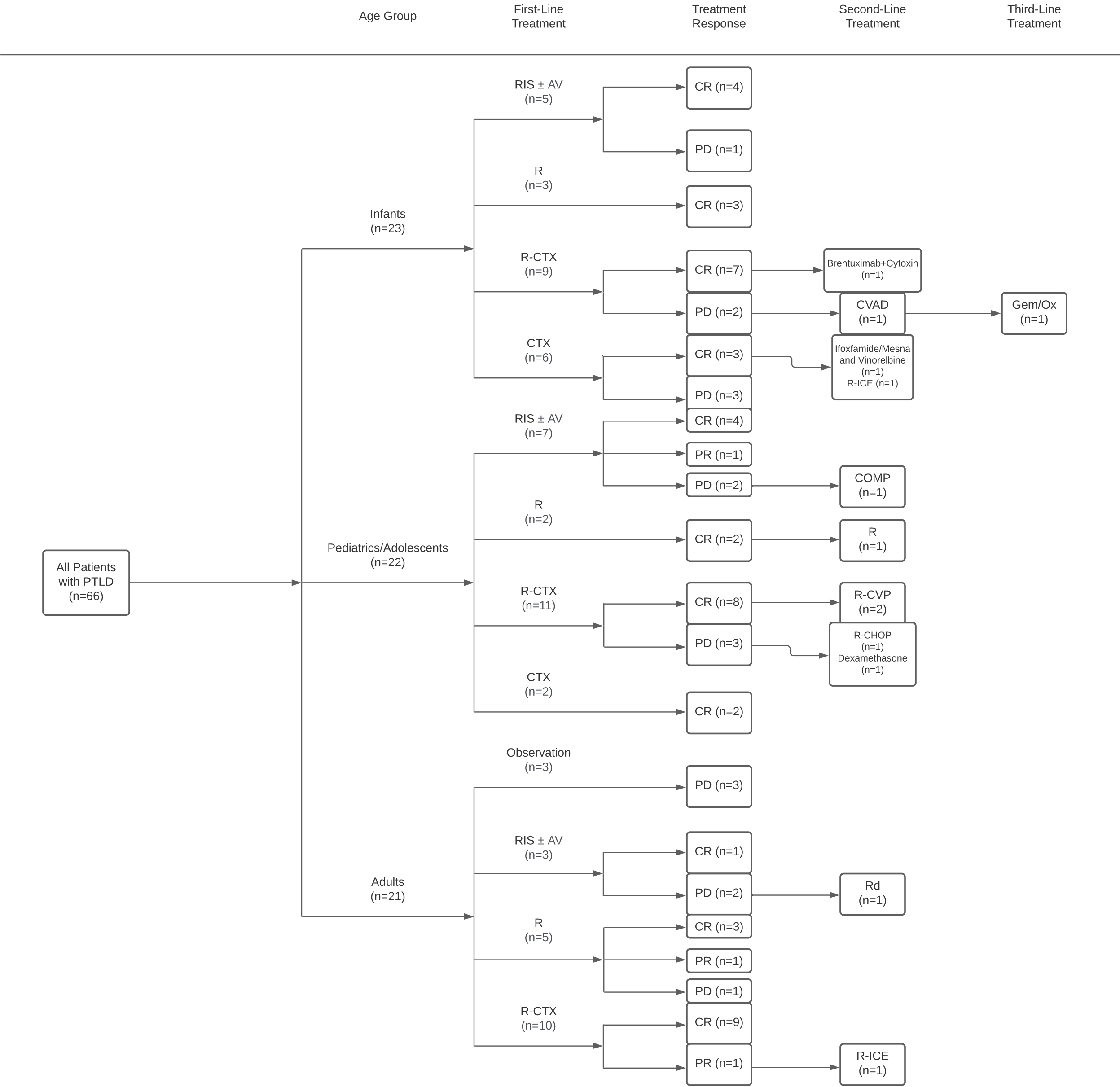

Supplement: Supplementary file 1 [file cancers-13-00899-s001.zip › Figure S2 - Treatment response for patients with any PTLD, stratified by age.pdf]

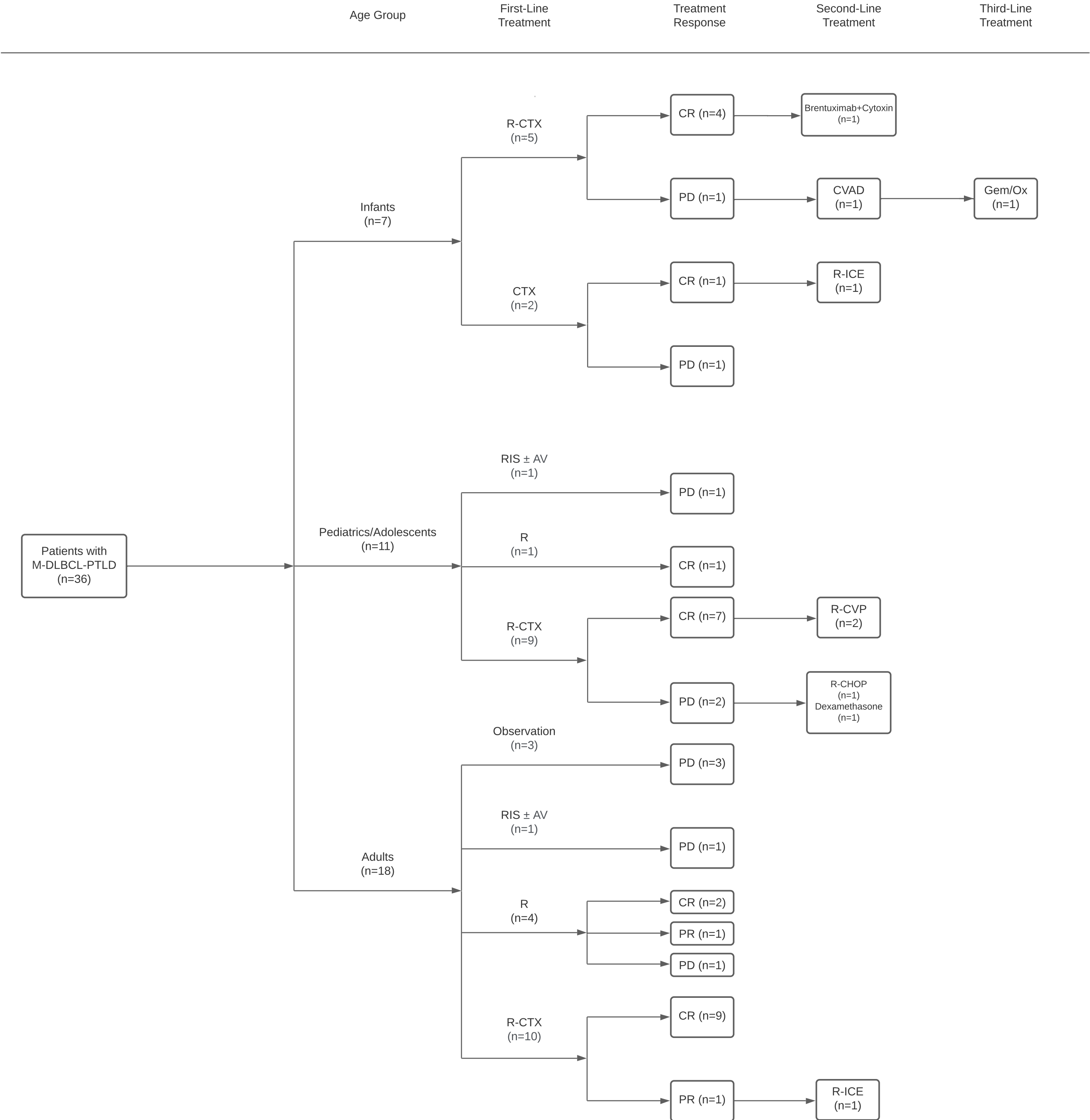

Supplement: Supplementary file 1 [file cancers-13-00899-s001.zip › Figure S3 - Treatment response for all patients with Monomorphic DLBCL-PTLD, stratified by age.pdf]

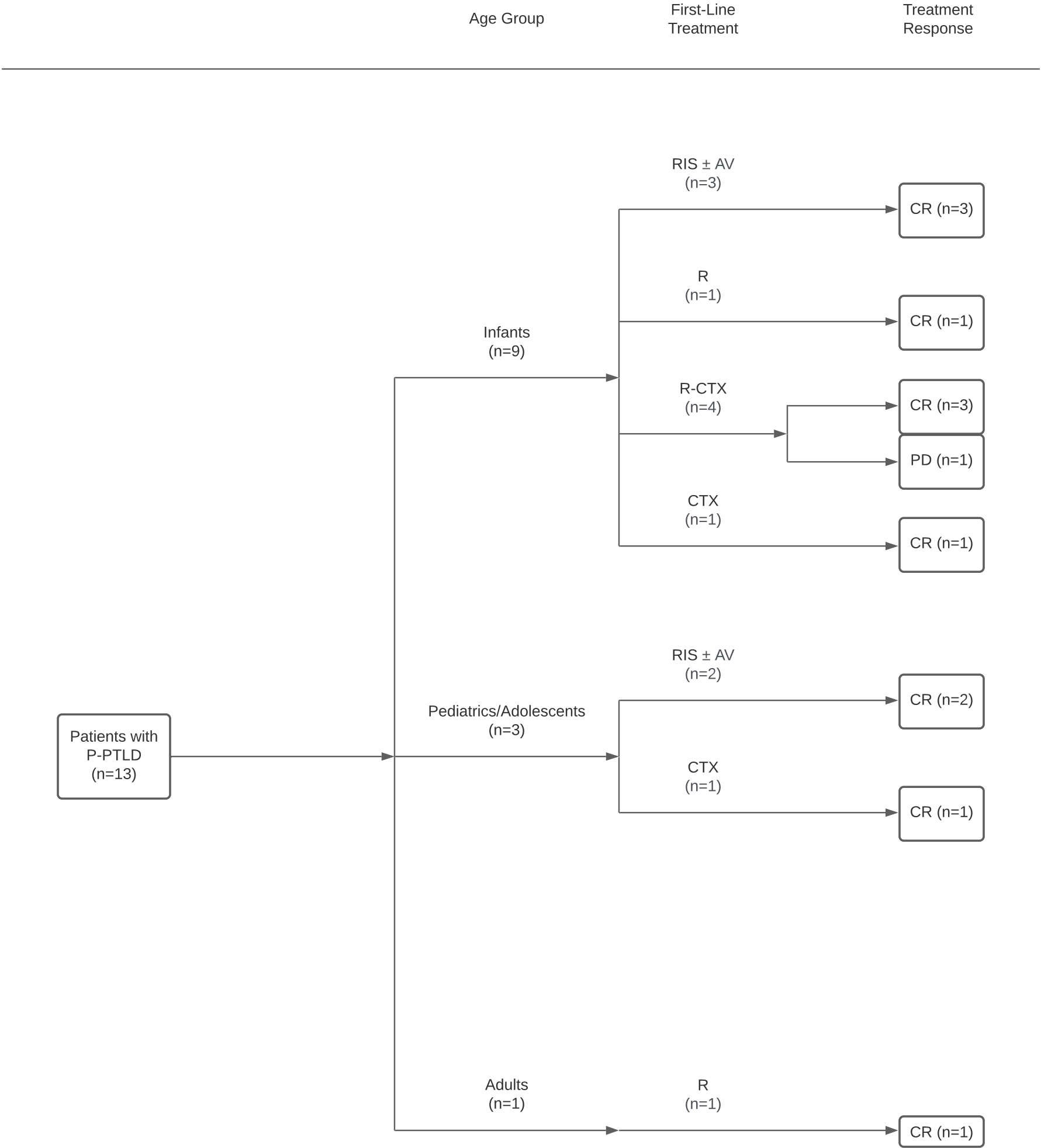

Supplement: Supplementary file 1 [file cancers-13-00899-s001.zip › Figure S4 - Treatment response for patients with Polymorphic-PTLD, stratified by age.pdf]

Age Group

First-Line  
Treatment

Treatment  
Response

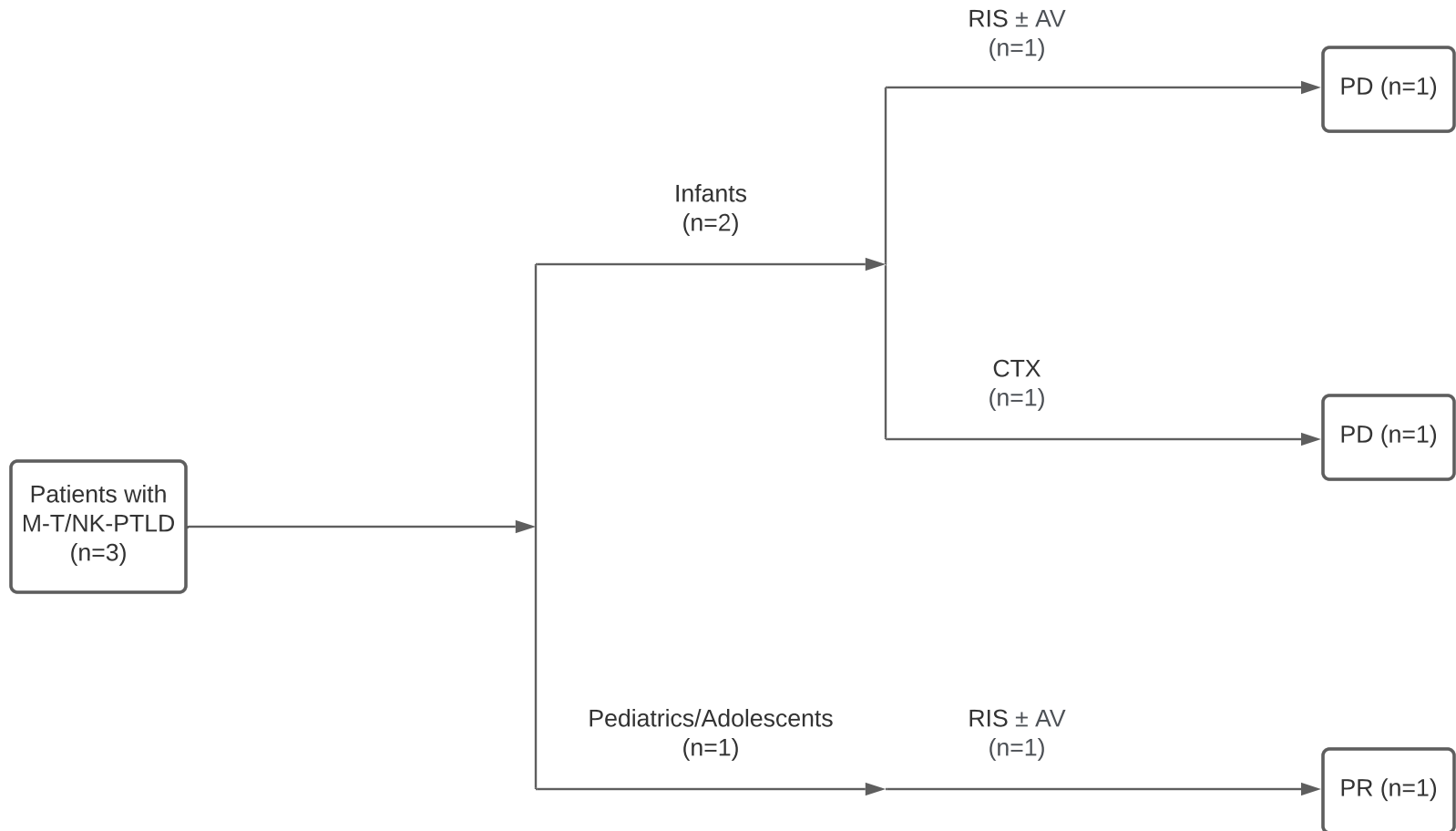

Supplement: Supplementary file 1 [file cancers-13-00899-s001.zip › Figure S5 - Treatment response for patients with TNK-PTLD, stratified by age.pdf]

Age Group

First-Line  
Treatment

Treatment  
Response

Second-Line  
Treatment

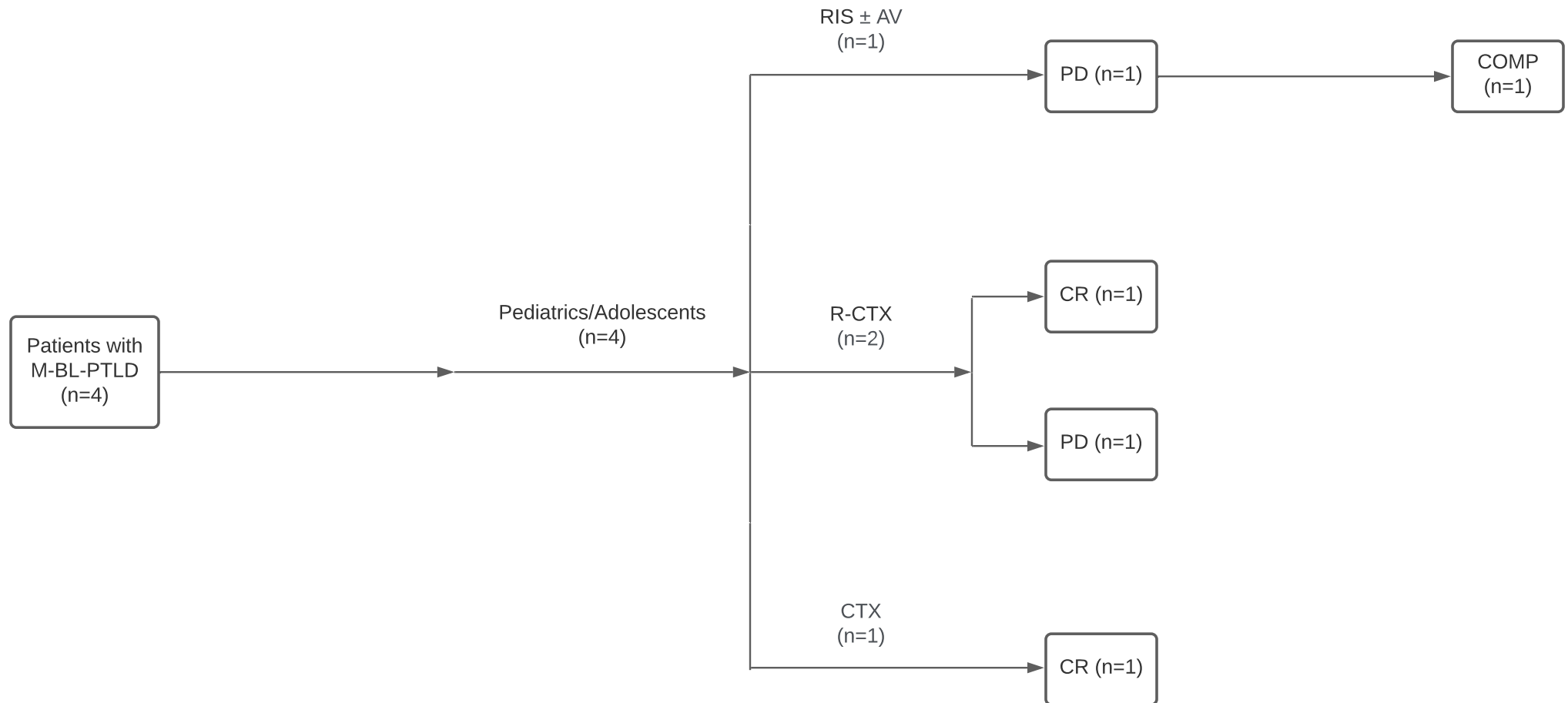

Supplement: Supplementary file 1 [file cancers-13-00899-s001.zip › Figure S6 - Treatment response for patients with Monomorphic Burkitt’s Lymphoma-PTLD, stratified by age.pdf]

Age Group

First-Line  
Treatment

Treatment  
Response

Second-Line  
Treatment

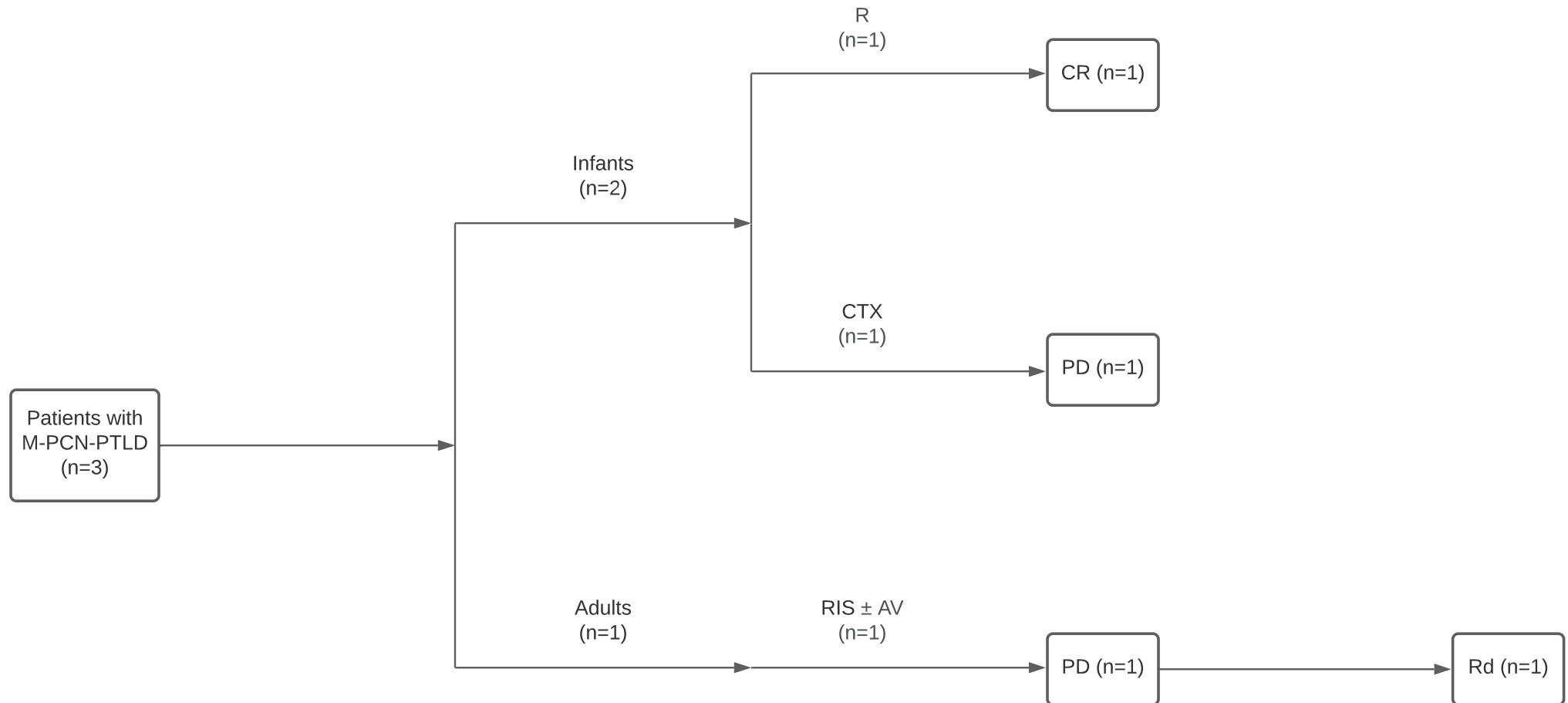

Supplement: Supplementary file 1 [file cancers-13-00899-s001.zip › Figure S7 - Treatment response for patients with Monomorphic Plasma Cell Neoplasm-PTLD, stratified by age.pdf]

Age Group

First-Line  
Treatment

Treatment  
Response

Second-Line  
Treatment

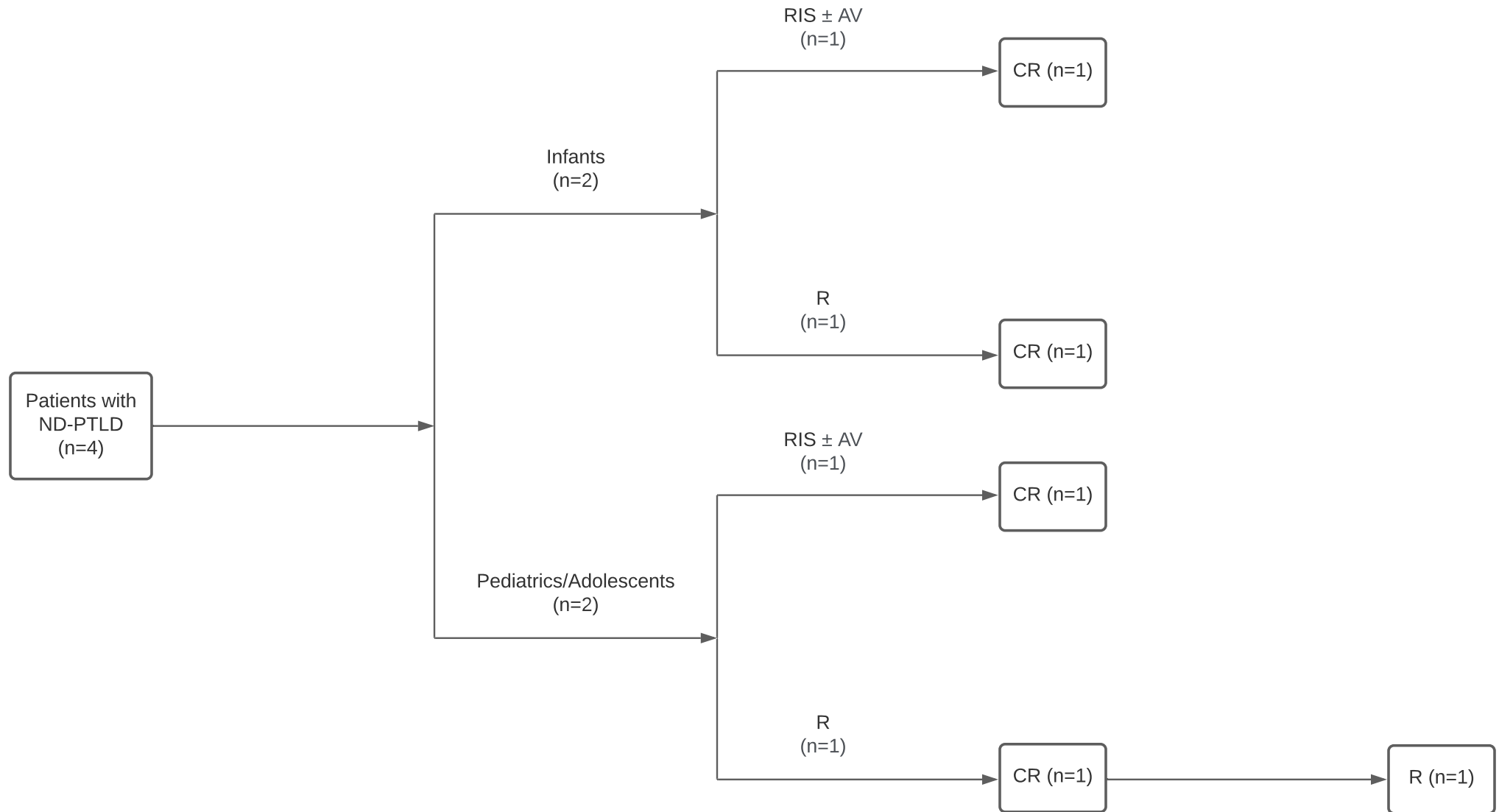

Supplement: Supplementary file 1 [file cancers-13-00899-s001.zip › Figure S8 -Treatment response for patients with Non-Destructive-PTLD, stratified by age.pdf]

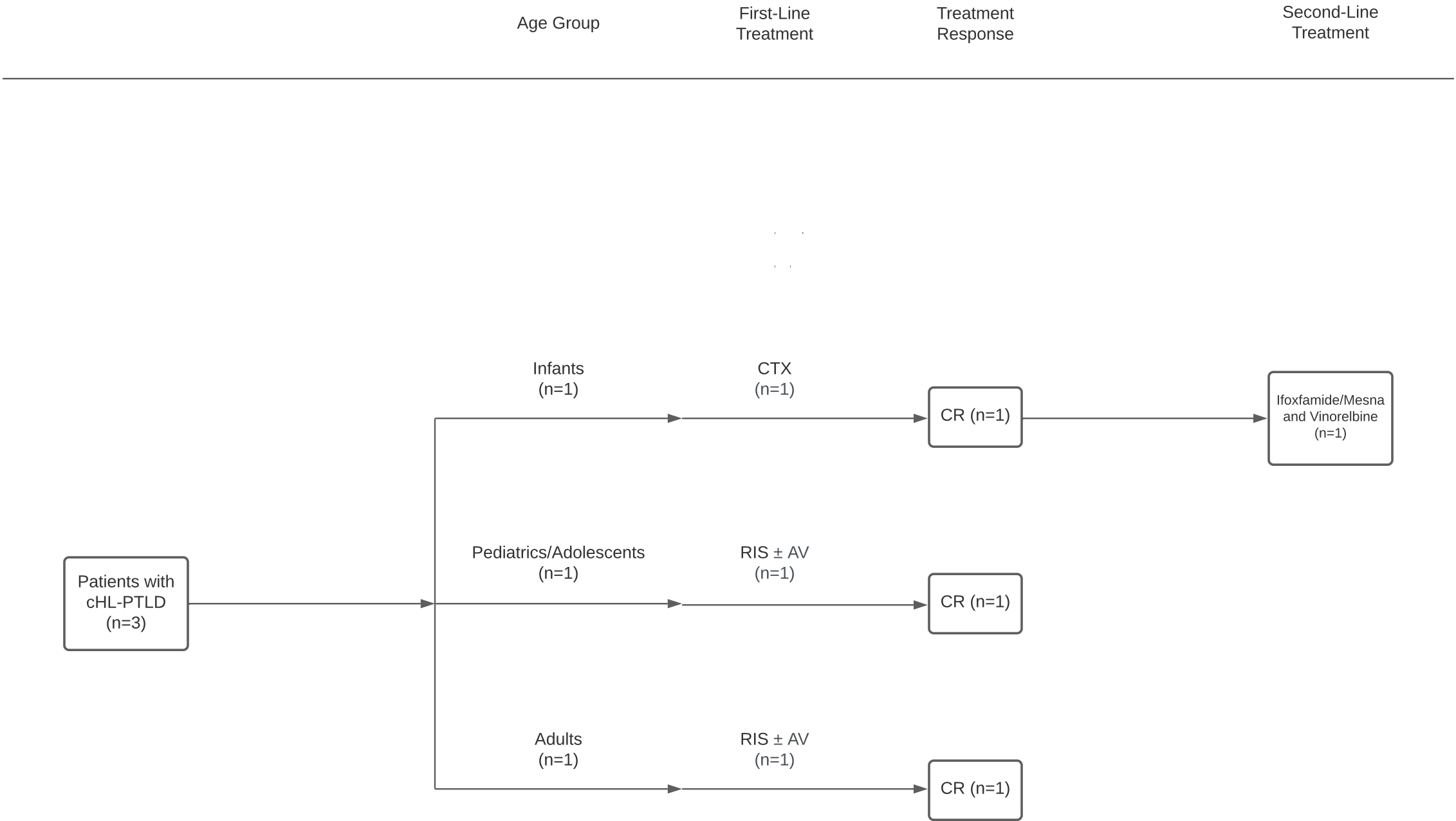

Supplement: Supplementary file 1 [file cancers-13-00899-s001.zip › Figure S9 - Treatment response for patients with Classical Hodgkin lymphoma-type PTLD, stratified by age.pdf]
